# Cigarette Smoking Is Associated with Prolongation of the QTc Interval Duration in Patients with Type 2 Diabetes Mellitus

**DOI:** 10.1155/2013/329189

**Published:** 2013-04-24

**Authors:** Petros Thomakos, Stavros Liatis, Stavroula Kalopita, Ioannis Vlahodimitris, Chryssoula Stathi, Nicholas Katsilambros, Nicholas Tentolouris, Konstantinos Makrilakis

**Affiliations:** First Department of Propaedeutic Medicine, Athens University Medical School, Laiko General Hospital, 17 Agiou Thoma Street, 11527 Athens, Greece

## Abstract

*Aims*. Aim of the study was to evaluate the effect of smoking on autonomic nervous system (ANS) activity and QTc interval duration in patients with Type 2 diabetes mellitus (T2DM). *Methods*. A total of 70 patients with T2DM (35 chronic smokers, 35 nonsmokers) treated with oral antidiabetic medications underwent continuous ECG Holter monitoring for 24 hours and analysis of time- and frequency-domain measures of heart rate variability (HRV). HRV over short time was also assessed using the deep breathing test. In addition, baroreflex sensitivity (BRS) was evaluated using the spontaneous sequence method. The mean QTc interval was measured from the 24-hour ECG recordings. *Results*. Smokers had lower body mass index (BMI) and exhibited higher 24-hour mean heart rate. There was no difference regarding all measures of ANS activity between the two groups. Smokers showed increased mean QTc duration during the 24 hours (439.25 ± 26.95 versus 425.05 ± 23.03 ms, *P* = 0.021) as well as in both day (439.14 ± 24.31 ms, *P* = 0.042) and night periods (440.91 ± 32.30 versus 425.51 ± 24.98 ms, *P* = 0.033). The association between smoking status and mean QTc interval persisted after adjusting for BMI. *Conclusions*. Cigarette smoking is associated with prolongation of the QTc interval in patients with T2DM by a mechanism independent of ANS dysfunction.

## 1. Introduction

Patients with Type 2 diabetes mellitus (T2DM) who smoke are at increased risk of developing cardiovascular disease, including cardiac arrhythmias [1]. Prolongation of the QT interval corrected for heart rate (QTc) is associated with a lowered ventricular fibrillation threshold and other potentially lethal arrhythmias, as polymorphic ventricular tachycardia (torsades de pointes), and has been proven to be an independent risk factor for sudden cardiac death (especially QTc values >440 ms), both in the general population [2] as well as in patients with T2DM [3].

Autonomic nervous system (ANS) function has been consistently shown to be associated with QTc interval duration in patients with diabetes and in healthy individuals [4]. Furthermore, in patients with diabetes, QTc interval prolongation is one of the main manifestations of cardiac autonomic neuropathy (CAN) [5]. There is no widely accepted single approach to the diagnosis of CAN in diabetes. Assessment of heart rate variability (HRV), orthostatic hypotension, and 24 h blood pressure profiles provides indices of both parasympathetic and sympathetic autonomic function and can be used in clinical settings. The analysis of HRV and measurement of arterial baroreflex sensitivity (BRS) have been proven to detect ANS dysfunction at a very early stage in patients with diabetes [5]. Furthermore, the evaluation of HRV during deep breathing is a highly sensitive and reliable test for early detection of parasympathetic dysfunction in a wide range of autonomic disorders [6]. Other methods, such as the classic battery of the tests of Ewing, cardiac sympathetic imaging, microneurography, and occlusion plethysmography, may also be used, especially in research settings [5].

It is well known that cigarette smoking alters autonomic cardiac control [7] and arterial baroreceptor function [8] and has also been demonstrated to prolong QT interval in healthy individuals in some [9–11] but not all studies [12]. However, there is relatively little information in the literature as regards to the impact of smoking on ANS function in general [13], and particularly on QTc interval duration in patients with T2DM, since both diabetes and cigarette smoking are considered as important modifiable risk factors for heart disease [1].

The aim of the present study was to evaluate QTc interval duration in smokers compared to nonsmokers with Type 2 diabetes mellitus, in relation to ANS activity.

## 2. Materials and Methods

### 2.1. Study Sample

A total of 35 consecutive chronic smokers with T2DM, matched one-to-one for age, sex, and duration of diabetes with 35 never-smokers with T2DM, were included in the study. To decide about the required sample size and since there is lack of relevant data in the literature, we, a priori, hypothesised that a QTc difference of 10 ± 5 ms between the two studied groups would be clinically significant and meaningful. Thus, and in order to achieve statistical power greater than or equal to 90%, at 5% Type I error rate of two sided hypotheses, a total of 35 consecutive chronic smokers with T2DM, matched one-to-one for age, sex, and duration of diabetes with 35 never-smokers with T2DM, was deemed adequate. Smoking status was expressed using the Brinkman index (BI), which was calculated by multiplying the number of cigarettes smoked per day by the duration of smoking in years [14]. None of the patients was taking insulin, *β*-blockers, antiarrhythmic drugs, and medications known to affect ANS activity or increase QT interval duration. In addition, patients who had coronary heart disease, cardiac arrhythmias, or any history of other chronic disease were excluded from the study. All patients had normal thyroid function and serum electrolytes. Patients who reported any hypoglycaemic event during the 24 hours prior to the study were also excluded. Hypertension was defined as systolic blood pressure ≥140 mmHg, diastolic blood pressure ≥90 mmHg, or the use of antihypertensive medication. The study was approved by the participating Hospital's Ethics Committee and carried out in accordance with the principles of the Declaration of Helsinki, as revised in 2008 [15]. The patients who took part in the study were attending the outpatient diabetes clinic of the Laiko General Hospital in Athens, Greece, and gave their written informed consent for participation. All smokers were encouraged to quit smoking.

### 2.2. Analysis of the 24-Hour Ambulatory ECG Recordings

All patients underwent continuous ECG Holter monitoring for 24 hours. For this study the digital ECG Holter recorder Spider View (ELA Medical, France) with seven electrodes was used to record three-channel ECGs. The 24-hour recordings were analysed using the SyneScope Holter analysis software (version 3.00, ELA Medical, France). Artefacts and ectopic beats were automatically edited from analysis. In addition, the QRS complex classification was reviewed by an experienced cardiologist blinded to the patients' clinical characteristics.

All the time- and frequency-domain parameters of HRV recommended by the Task Force of the European Society of Cardiology and the North American Society of Pacing and Electrophysiology were calculated from the 24-hour ECG recordings [16]. The values of the time-domain parameters of HRV were expressed in milliseconds (ms). HRV in the frequency-domain was computed by SyneScope using fast Fourier transformation analysis. The total power and, respectively, the power in very low frequency ((VLF) ≤0.04 Hz), low frequency ((LF), 0.04–0.15 Hz), and the high frequency ((HF), 0.15–0.40 Hz) bands were evaluated. All the frequency-domain parameters of HRV were calculated as absolute values and expressed in ms^2^. Furthermore, the LF and HF powers were expressed in normalised units (nu). The LF and HF powers expressed in nu represent the relative value of each power component in proportion to the total power minus the VLF component [16].

QT intervals were measured from the 24-hour ECG recordings using the semiautomated method and corrected for heart rate using Bazett's formula (QTc = QT/√RR) [17]. The QTc interval during the day and night periods was separately analysed (day period was defined as 9.00 am–9.00 pm; night period: 11.00 pm–6.00 am).

### 2.3. Evaluation of HRV during Deep Breathing and BRS Estimation

All measurements took place between 8:00 and 10:00 am in a quiet room, with stable temperature (20–24°C). Both tests were performed following an adequate rest period of 20 minutes. All patients were fasted for 8 hours and studied in the supine position.

BRS estimation was carried out by the spontaneous sequence method using the BaroCor System (AtCor Medical, Sydney, Australia). The BaroCor System enables the calculation of BRS from the estimated central blood pressure changes on heart rate using a radial artery tonometer (CBM 7000; Colins Medical Instruments Corp., San Antonio, TX, USA). Central blood pressure values were estimated from radial measurements using the mathematical transfer function proposed by Chen et al. [18]. Continuous ECG and blood pressure measurements were performed simultaneously for 20 minutes. Baroreflex effectiveness index (BEI) was also assessed through BaroCor System software. BEI quantifies the number of times in which the baroreflex is effective in driving the sinus node [19].

HRV during deep breathing was evaluated in all patients using the VariaCardio TF4-System (Medical Research Limited, Leeds, UK). The result provided by the software was assessed by calculating the ratio of the maximum and minimum heart rates during six cycles of paced deep breathing and expressed as the Expiration-Inspiration ratio (E/I ratio) [5].

### 2.4. Statistical Analysis

Continuous variables are presented as mean ± one-standard deviation. Normality of distributions was evaluated with the Kolmogorov-Smirnov test, and a significance level <0.05 was used to reject the null hypothesis of normal distribution. Nonnormally distributed variables were log-transformed for analysis. Comparisons between normally distributed continuous variables were performed with the calculation of the Student's *t*-test, while nonparametric variables with the Wilcoxon Mann-Whitney *U* Test. Associations between categorical variables were tested with the use of contingency tables and the calculation of the Chi-square test. Multivariate linear regression analysis was performed to assess the possible combined influence of the different variables on QTc interval duration. All statistical tests were performed by using SPSS v18.0 (SPSS Inc., Chicago, IL, USA).

## 3. Results

The demographic and clinical characteristics of the study participants are summarised in Table 1. Smokers and nonsmokers were not different regarding age, diabetes duration, HbA1c, fasting glucose, total cholesterol, triglycerides HDL-cholesterol, LDL-cholesterol, and blood pressure, but smokers had lower BMI (mean ± SD) (29.8 ± 5.1 versus 32.6 ± 5.2 kg/m^2^, *P* = 0.026). In addition there was no difference between the two groups regarding the use of medications for treatment of T2DM, hypertension, and dyslipidaemia. As expected, smokers had significantly higher mean 24-hour heart rate (80.4 ± 10.8 versus 73.1 ± 10.1 beats/min, *P* = 0.006), due to smoking-induced adrenergic stimulation.

All values of HRV parameters are presented in detail in Tables 2 and 3. As noted, there was no difference regarding all the time- and frequency-domain HRV measurements between the two groups. Furthermore, the BRS measurements and BEI did not differ between smokers and nonsmokers, and the E/I ratio was similar in both groups (Table 4).

**Figure 1:**
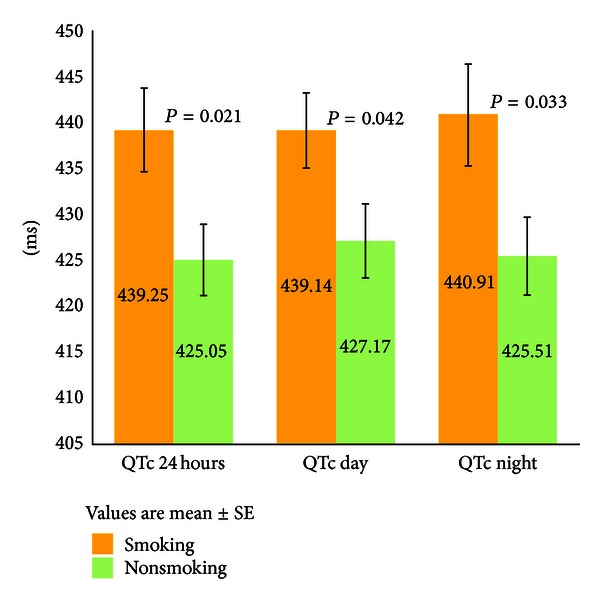
Results of mean QTc interval during the 24-hour, day, and night period in relation to smoking status.

Smokers showed increased mean QTc duration during the 24 hours (439.25 ± 26.95 versus 425.05 ± 23.03 ms, *P* = 0.021), as well as in both day (439.14 ± 24.31 versus 427.17 ± 23.99 ms, *P* = 0.042) and night periods (440.91 ± 32.3 versus 425.51 ± 24.98 ms, *P* = 0.033) (Figure 1, Table 4). Moreover, the association between smoking status and mean QTc interval during 24 hours and day and night periods remained significant after adjusting for BMI. Specifically, after adjusting for BMI, cigarette smoking was positively correlated with QTc during the 24 hours (*β* = 0.30, *P* = 0.015), as well as during the day (*β* = 0.27, *P* = 0.034) and night periods (*β* = 0.32, *P* = 0.015). However, there was no statistically significant correlation between mean QTc interval duration and the Brinkman index.

Furthermore, during the 24-hour period, 17/35 (48.5%) chronic smokers versus 10/35 (28.5%) nonsmokers (*P* = 0.086) exhibited QTc values longer than 440 ms (and, resp., 16/35 (45.7%) versus 11/35 (31%) (*P* = 0.22) during the day and 19/35 (54%) versus 11/35 (31%) (*P* = 0.064) during the night periods). 

## 4. Discussion

The present study exhibited a positive association between mean QTc interval duration and cigarette smoking in patients with Type 2 diabetes, which was evident also separately during both the day and night periods. To the best of our knowledge this is the first study in the literature to demonstrate such an association between cigarette smoking and QTc interval prolongation specifically in patients with T2DM.

Previous reports have provided conflicting data on the effect of smoking on QTc interval duration in healthy individuals. This may be due to numerous uncontrolled variables in the few published studies, such as the number of cigarettes smoked per day, tar and nicotine content of the cigarettes, personality factors, baseline sympathetic values, and the like [20]. Some studies showed prolongation of the QTc interval in smokers [9–11], while others did not [12]. Specifically, Ileri et al. [9] in a sample of 60 healthy volunteers (50% heavy smokers) reported that QTc was significantly longer in smokers compared to nonsmokers. This study was criticised [20] for possibly showing more acute than chronic effects of smoking on the results. Furthermore, Dilaveris et al. [10] disclosed in a sample of 1394 healthy subjects that only marginally prolonged QTc in smokers was seen, and Fauchier et al. [11] found that, among men who smoked, the number of cigarettes smoked per day was positively related to the corrected QT duration after adjustment for age. Contrary to these results, Mestre et al. [12], in a group of 37 smokers and 60 nonsmokers, found no significant differences in the duration of the QTc interval between smokers and nonsmokers.

Although there are many reports suggesting that smoking reduces HRV and attenuates BRS in normal individuals [7], there is only one study focusing on the effect of smoking on ANS activity in patients with diabetes, albeit without assessing QTc interval duration [13]. In that study, performed on 52 Japanese patients with T2DM, smokers exhibited lower BRS, lower myocardial uptake, and enhanced clearance of ^123^I-metaiodobenzylguanidine (^123^I-MIBG) compared to never-smokers, suggesting impaired ANS activity in the former group. HRV indices (namely, HF power and LF : HF ratio) were not different between the two groups. The distinction however of the present study was that BRS was assessed by the phenylephrine method, and HRV analysis was performed on 5 min ECG recordings. In the present study, BRS was assessed by the spontaneous sequence method, and HRV was measured by 24-hour ECG recordings, which are considered as more reliable techniques [16]. The association of cigarette smoking with QTc prolongation was found to be independent of ANS function in the current study. All cardiovascular ANS tests performed on the two groups did not reveal any association between smoking status and ANS measures. Specifically, the results of time- and frequency-domain analyses of HRV over a 24-hour period, heart rate response to deep breathing and BRS, were not significantly different in chronic smokers compared to nonsmokers. Furthermore, despite the fact that chronic smokers exhibited a significant increase in mean 24-hour heart rate, there was no difference in baseline blood pressure between the two groups, possibly due to the antihypertensive medications used.

There is evidence that, in patients with diabetes, QTc interval prolongation could be associated with an increased risk of unexpected death, while at the same time QTc has been shown to be an accurate predictor of cardiac death in newly diagnosed patients with T2DM [3]. In the Rotterdam QT Project, studying 6,693 patients, it was shown that prolonged mean QTc duration of greater than 440 ms over 24 hours was related to a 2.3-time higher risk for sudden death compared with a QTc of 440 msec or less [21]. The MONICA/KORA Augsburg Cohort Study [22], evaluating the predictive role of prolonged QTc on mortality in 160 patients with diabetes, concluded that QTc prolongation >440 ms is associated with a threefold increased mortality risk over 9 years but was weakly associated with cardiac autonomic neuropathy. Furthermore, prolongation of the QTc interval, even within the normal range, has been linked to increased cardiovascular risk [23]. In the present study, during the 24-hour period, there was a tendency for chronic smokers versus nonsmokers (*P* = 0.086) to exhibit QTc > 440 ms and, respectively, during the day (*P* = 0.22) and the night periods (*P* = 0.064).

There is no widely accepted single approach to the evaluation of ANS function in diabetes. Many methods have been proposed [5], a multitude of which were used in the current study. HRV assessment is considered a very valuable tool for the investigation of the sympathetic and parasympathetic functions of ANS [16]. In patients with diabetes, HRV evaluation derived from 24-hour ECG recordings has been proven to be more sensitive in detecting autonomic neuropathy than traditional autonomic reflex tests [5]. BRS assessment through the evaluation of the estimated central blood pressure changes on heart rate has been described to be more accurate in comparison to the peripheral blood pressure measurement [24]. The deep breathing test (expressed by the E/I ratio) is very easy to use and is considered as the most reproducible of the cardiac autonomic function tests [25]. A decreased heart rate variation in response to deep breathing has been suggested as a primary indicator of parasympathetic dysfunction [26]. Cigarette smoking stimulates the sympathetic nervous system mainly through the release of catecholamines by the adrenal cortex, and it has been demonstrated that in regular smokers the sympathetic nervous system is activated during the whole 24-hour period [27]. The increased sympathoadrenal activity has been shown to influence QT interval [4] and can potentially trigger malignant arrhythmias. In patients with T2DM, BRS is found to be negatively correlated with the QTc interval [28].

Strength of the present study is the fact that ANS function was assessed by a multitude of methods (HRV during 24 hours with continuous ECG recording, short-term HRV during deep breathing, and BRS) and thus could provide an accurate assessment of it. In addition, smokers were matched one-to-one to nonsmokers in terms of age, gender, and diabetes duration and thus could eliminate potential bias due to these confounding factors.

### 4.1. Potential Limitations

Cigarette smoking, especially in combination with T2DM, is well known to increase the risk for accelerated atherosclerosis, which leads to coronary artery disease [1]. Endothelial damage, increased oxidative stress, exposure to chronic inflammation, impaired endogenous fibrinolysis, increased thrombosis susceptibility, and formation of advanced glycation end products (AGEs) mediated by smoking may all have an additional role to the QTc interval prolongation associated with T2DM [1]. The present study, being a retrospective cohort one, was not able to investigate this possible effect of cigarette smoking on CVD risk through QTc prolongation in diabetic patients, which should be assessed in a different study.

Furthermore, it is very difficult to deduce that there is a cause and effect connection between QTc interval prolongation and smoking from the present data. Subclinical coronary heart disease, known to prolong QTc interval [29], cannot be definitely ruled out, since coronary heart disease was excluded only on the premises of ECG findings and clinical history. It also remains controversial whether the effect of smoking on QTc interval prolongation is mostly an acute or a chronic event, since we have observed a greater mean QTc interval in smokers during the night (440.91 ± 32.3) compared to the day periods (439.14 ± 24.31) when participants were apparently more likely to smoke. Maybe this finding could be explained by the fact that QTc interval shows diurnal variation, since it has been shown that, in people with normally innervated hearts, QTc intervals are longer during sleep than during waking hours [30]. Finally, the present study could not provide any answer to the question of whether or not there is a linear dose effect of smoking on QTc interval duration (the BI was not associated with QTc duration). This finding can be explained by the fact that the number of patients studied was relatively small in order to evaluate this effect of the amount of smoking on QTc duration.

## 5. Conclusion

In conclusion, the present study showed that cigarette smoking was associated with prolongation of the QTc interval in patients with T2DM by a mechanism independent of ANS dysfunction, and this effect may be implicated with an increased cardiovascular risk in this population. Confirmation of the current findings in a larger prospective cohort study is definitely needed in order to validate these results and assess their generalizability in the general diabetic population.

## Figures and Tables

**Table 1:** Patients' demographic and clinical characteristics (data are expressed as mean ± SD).

Variable	Chronic smokers	Nonsmokers	*P*
*N*	35	35	
Gender F-M	15-20	15-20	
Age (years)	55.1 ± 9.0	56.9 ± 8.2	0.379
Body mass index (kg/m^2^)	29.8 ± 5.1	32.6 ± 5.2	**0.026**
Heart rate (beats/min)	80.4 ± 10.8	73.1 ± 10.1	**0.006**
SBP (mmHg)	125.3 ± 14.6	128.1 ± 15.6	0.442
DBP (mmHg)	74.2 ± 10.3	77.1 ± 8.5	0.205
Total cholesterol (mmol/L)	5.0 ± 0.9	4.7 ± 1	0.302
Triglycerides (mmol/L)	2.0 ± 1.9	1.8 ± 0.8	0.700
HDL-C (mmol/L)	1.1 ± 0.3	1.2 ± 0.3	0.366
LDL-C (mmol/L)	3.1 ± 0.9	2.7 ± 0.9	0.066
Fasting glucose (mmol/L)	7.4 ± 2.2	7.3 ± 1.5	0.788
HbA1c (mmol/mol)	55.2 ± 11.5	51.3 ± 10.3	0.145
Duration of diabetes (yrs)	5.3 ± 4.5	5.3 ± 4.3	0.936
Brinkman index (cigarettes/day × years)	927 ± 735	—	
Number of patients with arterial hypertension	12	17	0.225
Number of patients on ACE inhibitors	7	10	0.403
Number of patients on AT1 antagonists	7	9	ns
Number of patients on ACE + AT1	1	1	ns
Number of patients on Ca-blockers	3	5	ns
Number of patients on diuretics	7	7	ns
Number of patients on metformin	31	28	ns
Number of patients on sitagliptin	14	8	0.122
Number of patients on sulfonylureas	10	16	0.138
Number of patients on glitazones	6	10	ns
Number of patients on meglitinides	2	0	ns
Number of patients on statins	14	19	0.231

SBP: systolic blood pressure; DBP: diastolic blood pressure; HDL-C: high density lipoprotein cholesterol; LDL-C: low density lipoprotein cholesterol; ACE: angiotensin-converting enzyme.

Hypertension was defined as systolic blood pressure >140 mmHg or diastolic blood pressure >90 mmHg or the use of antihypertensive medication.

**Table 2:** Mean values (±SD) of frequency-domain parameters of HRV during 24-hour, day, and night periods.

Variable	Chronic smokers	Nonsmokers	*P*
Total power 24 hours (ms^2^)	2339 ± 1864	2242 ± 1212	0.800
Total power day (ms^2^)	1984 ± 1570	1768 ± 894	0.497
Total power night (ms^2^)	2734 ± 2733	2800 ± 1877	0.908
Low frequency (LF) power 24 hours (ms^2^)	585 ± 684	561 ± 436	0.862
LF power 24 hours in normalised units (nu)	64.36 ± 11.79	65.87 ± 11.02	0.590
LF power day (ms^2^)	482 ± 531	428 ± 307	0.616
LF power day (nu)	63.46 ± 9.45	65.74 ± 11.57	0.389
LF power night (ms^2^)	768 ± 1186	754 ± 762	0.955
LF power night (nu)	66 ± 14.01	66.76 ± 11.40	0.809
High frequency (HF) power 24 hours (ms^2^)	208 ± 374	181 ± 200	0.707
HF power 24 hours (nu)	19.39 ± 7.79	19.28 ± 8.37	0.956
HF power day (ms^2^)	170 ± 295	114 ± 140	0.331
HF power day (nu)	17.89 ± 7.74	16.27 ± 6.78	0.374
HF power night (ms^2^)	284 ± 576	257 ± 309	0.815
HF power night (nu)	21.26 ± 10.17	21.1 ± 9.17	0.946
LF/HF ratio 24 hours	4.15 ± 2.64	4.24 ± 2.31	0.771
LF/HF ratio day	4.6 ± 3.03	5.01 ± 2.78	0.566
LF/HF ratio night	4.55 ± 3.95	3.91 ± 2.17	0.423

Day period: 09:00–21:00; night period: 23:00–06:00.

**Table 3:** Mean values (±SD) of time-domain parameters of HRV during 24-hour, day, and night periods.

Variable (ms)	Chronic smokers	Nonsmokers	*P*
SDNN 24 hours	114.98 ± 33.05	123.32 ± 39.04	0.348
SDNN day	92.64 ± 30.64	97.91 ± 26.15	0.452
SDNN night	83.43 ± 27.55	90.41 ± 30.68	0.331
PNN30 24 hours	13.79 ± 11.79	13.58 ± 10.99	0.94
PNN30 day	11.70 ± 11.45	10.06 ± 9.3	0.523
PNN30 night	17.03 ± 14.71	20 ± 15.81	0.43
RMSSD 24 hours	28.41 ± 17.28	28.51 ± 14.18	0.979
RMSSD day	26.51 ± 16.07	24.41 ± 12.36	0.553
RMSSD night	30.61 ± 22.3	34.01 ± 18.77	0.502
SDANN 5 min 24 hours	104.34 ± 31.65	112.5 ± 40.04	0.358
SDANN 5 min day	82.04 ± 30.4	86.97 ± 27.95	0.493
SDANN 5 min night	62.19 ± 20.94	67.69 ± 31.97	0.407

SDNN: standard deviation of all normal-to-normal RR intervals; PNN30: number of pairs of adjacent NN intervals differing >30 ms in the entire recording divided by the total number of all NN intervals; RMSSD: the square root of the mean of the sum of the squares of differences between adjacent NN intervals; SDANN: 5 min, standard deviation of the averages of NN intervals in all 5-minute segments of the entire recording.

**Table 4:** Mean values (±SD) of E/I index, BRS measurements, and QTc interval duration during 24-hour, day, and night periods.

Variable	Chronic smokers	Nonsmokers	*P*
E/I index	1.29 ± 0.15	1.23 ± 0.1	0.085
RBRS (ms mmHg^−1^)	9.35 ± 5.97	8.07 ± 3.73	0.287
CBRS (ms mmHg^−1^)	8.09 ± 4.57	7.19 ± 4.08	0.388
SE RBRS	0.99 ± 0.78	0.81 ± 0.64	0.308
SE CBRS	0.92 ± 0.87	0.73 ± 0.68	0.313
RBEI	0.294 ± 0.157	0.331 ± 0.121	0.283
CBEI	0.287 ± 0.162	0.327 ± 0.128	0.271
QTc 24 hours (ms)	439.25 ± 26.95	425.05 ± 23.03	**0.021**
QTc day (ms)	439.14 ± 24.31	427.17 ± 23.99	**0.042**
QTc night (ms)	440.91 ± 32.3	425.51 ± 24.98	**0.033**

E/I index: expiration/inspiration index; RBRS: radial baroreflex sensitivity; CBRS: central baroreflex sensitivity; RBEI: radial baroreflex effectiveness index; CBEI: central baroreflex effectiveness index.

CBRS was assessed through the evaluation of the estimated central blood pressure changes on heart rate.

RBRS was assessed through the evaluation of the radial artery blood pressure changes on heart rate.
